# The ManVan: a mobile cancer support service for men with prostate, testicular and penile cancer in Wales

**DOI:** 10.3332/ecancer.2015.603

**Published:** 2015-12-01

**Authors:** Rachel Iredale, Rhiannon Skilton, Richard Pugh, Heather Blake

**Affiliations:** 1 Tenovus Cancer Care, Cardiff, Wales, UK; 2 Prostate Cancer UK, London, UK; 3 University of South Wales, Pontypridd, Wales, UK

**Keywords:** prostate, testicular, penile, mobile, community, nurse specialist, counselling, welfare benefits

## Abstract

The ManVan commenced service delivery on 1st April 2014 and is the United Kingdom’s first dedicated mobile support service for men affected by prostate, testicular, and penile cancer. It is delivered in partnership with Prostate Cancer UK and Movember and fully funded by the Movember Foundation. It brings nursing care, counselling for individuals and couples, group support and welfare rights advice, directly to men living with prostate, testicular and penile cancer in communities across Wales. The ManVan has travelled extensively across Wales during its first year, visiting 94 different locations, across all seven Local Health Board areas.

The first half of the year began with a Roadshow where we welcomed thousands of visitors on board, including men and women worried by all sorts of cancer symptoms; their family and friends; health and social care professionals and politicians. The variety of venues the ManVan has visited has included hospitals, supermarkets, social clubs, caravan fairs and rugby grounds to help raise awareness of the ManVan service and identify potential clients. As expected the greatest proportion of visitors are male, particularly older men.

In the second half of the year, we focussed on our target audience – men diagnosed with prostate, testicular, or penile cancer and their families. Using a targeted approach to urology clinics across NHS Wales, as well as community-based activity encouraging men from ethnic minorities to visit, we have now taken on 161 clients, many of whom have received more than one ManVan service, and attended on more than one occasion. Most of our clients have prostate cancer, are over the age of 55 years, and are married. Analysis of the early data around the clinical and psychosocial benefits of the services offered on the ManVan is positive and the annualised value of the benefits obtained for ManVan clients equates to more than £300,000 in this first year. There were 3,319 visitors to the ManVan in total during the first year.

## Introduction

About 50 people are diagnosed with cancer in Wales every day with more than 18,000 new cases of cancer diagnosed in 2012 [1]. The NHS in Wales currently spends about 7% of its health budget on cancer services [2]. Although this is a greater proportion of the health budget than is spent on cancer services in England, demand in Wales is also greater due to higher levels of cancer incidence. In 2011, the incidence of all cancers in England (excluding non-melanoma skin cancer) was 392.5 per 100,000 population, compared to a Welsh figure of 411.3 [3]. The quality and availability of cancer services across Wales varies considerably; some cancer specialities have excellent support services, while others have none. As people are living longer, cancer is increasingly being recognised as a long-term condition. Similarly, survivorship is becoming a key issue for patients, policy makers, health and social care practitioners and cancer charities. The latest survival figures reveal that approximately 50% of people survive cancer for 10 or more years in England and Wales [4]. As more and more people survive, there is a need to explore new models and approaches to care for those living with, and beyond, cancer.

According to the Welsh Cancer Intelligence and Surveillance Unit (2015) [1], there are almost 10,000 men in Wales diagnosed with cancer every year and prostate cancer accounts over a quarter of all these new cancers in men. Additionally, more than 500 men die every year from prostate cancer in Wales [5]. There are a high number of deprived areas in Wales [6], a tradition amongst some men in deprived areas of unhealthy diets and lifestyles [7], and a lack of awareness of key symptoms, issues which add to the reluctance of men to present their health problems to a doctor. Men with prostate, testicular and penile cancer are not well served. Inequalities exist between different Local Health Boards, especially in rural areas and these inequalities are not limited to clinical treatment. There is often little or no psychosocial support to help people cope at home once active cancer treatment has finished. Financial stability, psychological impact and sexual relationships are rarely considered, and limited support in these areas has a substantial impact on well-being.

### The ManVan

Due to this limited service provision and support, we decided to build a ManVan that was launched on 1 April 2014. The ManVan is the United Kingdom’s first dedicated mobile support service for men affected by prostate, testicular, and penile cancer. We know men are not always comfortable talking about their worries, so we designed a space specifically with them in mind. The aim of the ManVan is to provide a full range of survivorship services in a single vehicle and to take cancer support services to where they are needed most – into the heart of Welsh communities. The ManVan has been funded by The Movember Foundation through Prostate Cancer UK for a period of three years.

ManVan staff includes a logistics officer, who drives the vehicle, a number of specialist nurses, a counsellor, and a welfare rights advisor. The ManVan brings nursing advice, counselling for individuals and couples, group support and benefits advice directly to men living with these cancers to communities across Wales. Specific objectives are as follows:

- To promote a meaningful quality of life for men affected by prostate, testicular, and penile cancer through the provision of a suite of survivorship services
- To improve knowledge and understanding of the consequences/side effects of treatments
- To manage better the expectations men have after their diagnosis and treatment
- To increase access to counselling and emotional support services to improve both physical and mental well-being
- To enhance the quality of life of men surviving prostate, testicular and penile cancer and their families, carers, and friends.

The locations visited by the ManVan are determined by the location of urology clinics and the needs of new and existing clients. The ManVan location calendar is published on the Tenovus Cancer Care Website and distributed to a network of local contacts and newspapers on a monthly basis. We also distribute leaflets in local communities in places such as general practitioner (GP) surgeries and pharmacies, as well as making considerable use of social media, such as Facebook and Twitter, to promote where and when the ManVan will be.

Men are able to access the ManVan service in a number of various ways: they can book an appointment through the Tenovus Cancer Care Support Line; they can use an automated texting service to arrange a callback, or can simply drop into the ManVan when it is in their community. A referral from a healthcare professional is not necessary and every man can access these services for as long as he needs.

## Methods

Mobile units provide practical, patient-centred solutions to meeting the needs of cancer patients across Wales. Tenovus have been modelling this approach since 2009, when we launched a Mobile Cancer Support Unit for chemotherapy in partnership with Velindre Cancer Centre in South Wales [8]. The Mobile unit provides easy access to treatment, support and advice in a safe environment that is loved by patients and their families. It epitomises a new kind of cancer care allowing a range of services to be provided on board by integrating clinical, emotional, financial, practical, and supportive services.

As part of the ManVan service, we are collecting and analysing data internally about the following:

***1. Visitors:*** Defined as those dropping in but not using any services. Data are being collected from visitors by the way of a short feedback form that will be administered on the ManVan immediately following the visit. Visitor numbers vary considerably depending on the location of the ManVan.

***2. Patients/Clients:*** Defined as those using a service. This can be use of a single service or a number of services on an on-going basis. Data are collected at baseline using self-complete survey methods that includes sociodemographic data, clinical data, and satisfaction data. We are also using a series of psychometric scales to focus on therapeutic outcomes and measures of perceived social support/self-efficacy/well-being which will normally take place one month after the initial appointment for nursing advice, but more frequently for patients using psychological or financial support services. The timing can be adjusted or the frequency increased to take treatment or patient availability into account. The ManVan team have capacity to book up to 36 appointments each week with new or existing clients.

The scales used vary dependent on the service(s) received by the patient and include the following:

**EPIC-26:** The expanded prostate cancer index composite (EPIC) is a comprehensive instrument designed to evaluate patients after prostate cancer treatment [9]. EPIC has been validated in men with localised prostate cancer who undergo surgery, external beam radiation, or brachytherapy with or without the use of hormonal adjuvants. Every client who has a nursing appointment completes one EPIC-26 form on board the ManVan and one after four to six weeks over the phone with a ManVan nurse. All EPIC-26 scores are standardised on scale of 0–100 with higher scores representing better health-related quality of life results (HRQLR). All scores are grouped according to the five domains of EPIC-26 and one other relating to urinary incontinence, urinary irritative/obstructive, bowel, sexual, hormonal and general urinary function.

**Session evaluation:** The session evaluation questionnaire (SEQ) developed by Stiles (1980) to measure positivity and arousal is completed by all clients ‘before’ and ‘after’ their time on the ManVan [10]. The SEQ is a 7-point bipolar adjective scale capturing 10 recognised dimensions of mood and emotion [11]. Each item is scored from 1 to 7 and inverted as appropriate, with higher scores indicating greater positivity or arousal. Scores are calculated as the mean ratings on the appropriate items rather than the mean of the item scores so that the final score lies on the same 7-point scale as the individual items, for clearer interpretation. SEQ results are measured by visit not by person.

**Session impact:** In addition to the session evaluation questionnaire, the session impact scale (SIS) measuring depth and smoothness, is also completed immediately ‘after’ a client’s time on board the ManVan. The purpose of the SIS is to assess participant’s perception of the overall value of the session plus the perception of session ease. It is a 7-point bipolar scale measuring 11 dimensions of overall depth and smoothness [12]. A total of 161 clients completed or partly completed the SIS following their time on the ManVan. The most positive score a client can give for any of the 11 scale measures is 7 (smoother and with more depth) and the lowest is 1 (rougher and shallower).

**CORE-10:** CORE-10 is a measure of ‘psychological distress’ used by all Tenovus Cancer Care counsellors [13]. ManVan clients receive a CORE-10 form at ‘assessment’, ‘first’, ‘middle’, and ‘discharge’ session so CORE-10 form numbers will rise when clients start receiving their third counselling appointment. Items cover anxiety (2 items), depression (2 items), trauma (1 item), physical problems (1 item) functioning (3 items – day to day, close relationships, social relationships) and risk to self (1 item) [13]. CORE-10 scores are normally divided into four categories, as shown in Table 1.

***3. Service activity and performance metrics:*** Defined as data relating to the ManVan which is monitored on a monthly basis and which includes the following:

- distances travelled and locations visited
- numbers and demographic data of visitors
- availability and uptake of client appointments broken down by service, type of cancer, and by demographics

Finally, we are commissioning an independent external evaluation of the overall project which will be conducted by the Welsh Institute of Health and Social Care using a mixed methods approach. This aspect of the evaluation will explore the acceptability of the ManVan survivorship service to patients, family members, carers and to health and social care practitioners across Wales and whether the ManVan is a cost-effective solution that delivers a better experience and quality of care for men with prostate, testicular, and penile cancer across Wales.

## Results

**Figure 1.**
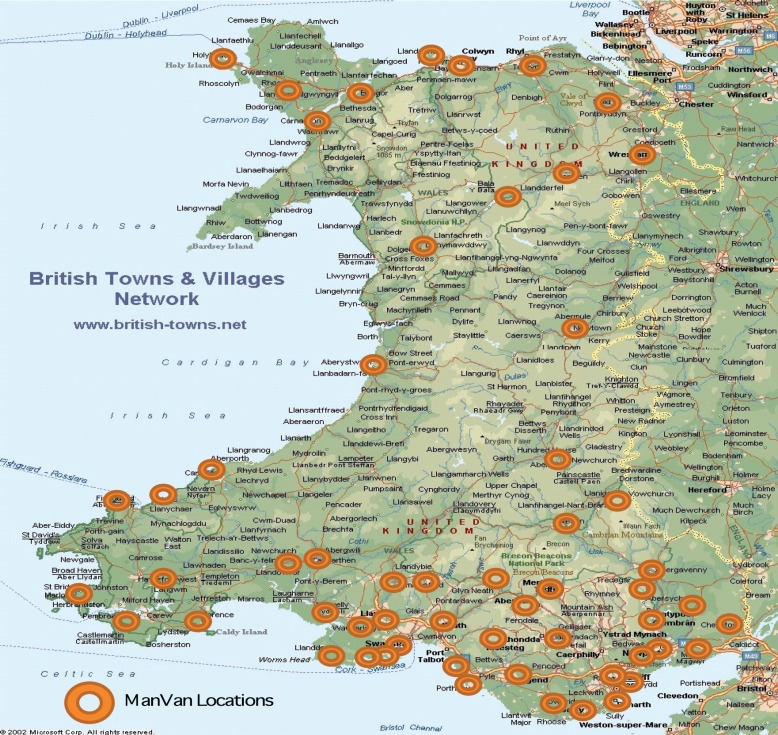
Map of Wales with locations visited.

The ManVan has travelled extensively across Wales during its first year, visiting a total of 94 different locations, across all seven Health Board Areas (see Figure 1). Over the year, we have travelled 10,923 miles using 12,960 litres of fuel. We have welcomed more than 3,300 visitors on board, including men and women worried by all sorts of cancer symptoms; their family and friends; health and social care professionals, and politicians. From Barry Island in South Wales to Bangor in North Wales, the variety of venues the ManVan visited has included hospitals, supermarkets, social clubs, caravan fairs, and rugby grounds to help raise awareness of the ManVan service and identify potential clients. As expected, the greatest number of visitors has been male, particularly older men.

Using a targeted approach to urology clinics across NHS Wales, as well as community-based activity encouraging men from ethnic minorities to visit, we have now taken on 161 clients, many of whom have received more than one ManVan service, and attended on more than one occasion. Most of our clients have prostate cancer, are over the age of 55 years, and are married. Analysis of the early data around the clinical and psychosocial benefits of the services offered on the ManVan is positive and the annualised value of the benefits obtained for ManVan clients have totalled more than £300,000 in this first year.

**Figure 2.**
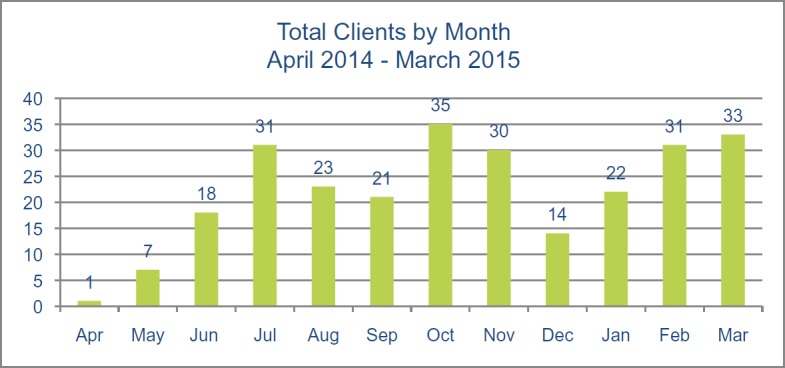
Total ManVan clients per month, 2014–2015.

Although the total number of unique clients for the year was 161, the total number of ManVan client visits for the year was 266. This illustrates that many clients utilised more than one ManVan service and are also being seen on board on more than one occasion. See Figure 2 for the number of clients seen on the ManVan every month during the first year.

Most of the ManVan clients are over the age of 55 (89%). The age range is 34–88 years and the mean is 67 years. This is largely due to the age of men diagnosed with prostate cancer. The largest numbers of ManVan clients live in the Hywel Dda Health Board area that covers West Wales. This is largely due to an effective partnership with urology clinicians at Prince Phillip Hospital in Llanelli who refer many of their patients to the ManVan. The ManVan team work closely with urology teams across Wales, the most successful partnerships occurring where existing services are under-resourced, and there is a clinician willing to act as an advocate for the service. Over 80% of the clients seen on the ManVan in the first year had prostate cancer, 2% had testicular cancer, and 2% had penile cancer. A small number of clients had another cancer, such as head and neck cancer or bladder cancer and were seen as they were experiencing issues similar to our clients, such as incontinence. The remaining clients are the spouses or carers of clients who have received services. The majority of clients we have seen this year normally travel between 11 and 20 miles to receive treatment for their cancer, and the majority have also found the reduction in travel and the multi-disciplinary nature of the ManVan to be more convenient or equal in convenience to use than their normal cancer care location. In addition, as the ManVan operates a drop-in service so men can re-access support whenever the vehicle visits their community.

The total number of appointments offered and attended is outlined in Table 2.

## Clinical outcomes: EPIC-26

**Figure 3.**
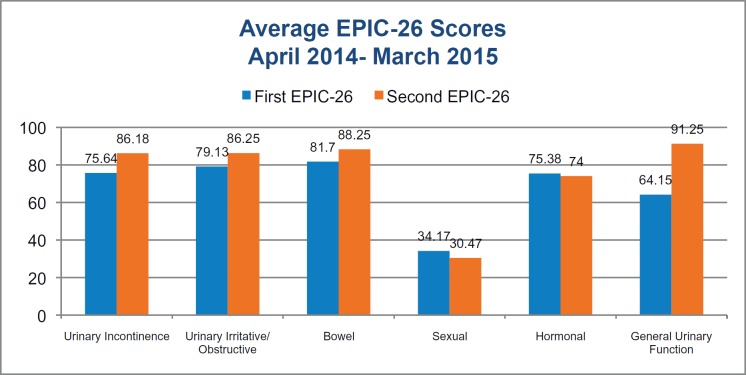
Average EPIC-26 scores at baseline and follow-up.

The results in Figure 3 show average scores for **53** clients who completed one EPIC-26 form, together with the **20** clients who completed a second form. At the first appointment, clients are scoring best in the bowel domain and worse in the sexual domain.

For this EPIC-26 scale, the averages from the two groups of men are not directly comparable because the number of people completing the second form is fewer than for the first form. We will be able to draw firmer conclusions from these data about the progress of ManVan clients as the volume of data collected increases over the next two years. However, these early results do demonstrate the degree of impact experienced by men undergoing prostate cancer treatment.

## Psychometric scales

### Session evaluation (SEQ)

While all clients are asked to complete a SEQ form the majority who agree to do so are currently those receiving Welfare Benefits advice. A total of 161 clients completed or partly completed a ‘before’ and ‘after’ SEQ on board the ManVan during the year. Tables 3 and 4 total average scores for every client and the change between ‘before’ and ‘after’ their time on the ManVan. The results are positive and show an overall increase in positivity and arousal (see Table 5).

Because each client is asked to complete this form upon arrival and departure the results can be directly correlated with the advice, support and information received on board.

### Session impact

The SIS data for the year show similar result on average for both depth and smoothness. Again these early results show an improvement.

The session evaluation questionnaire and session impact scale are used together to give an overall score for the four domains. When looking at the two sets of results, Smoothness scores highest and Arousal lowest. All four domains of mood and emotion are above the halfway score of 3.5 which, again, reflects positively on the ManVan.

### Core

A total of 22 clients have completed at least 1 CORE-10 form during the year, with 15 of these clients completing at least 2 forms. Table 6 shows the mean change in scores between stages.

There are at least four CORE-10 forms to complete with each client, and the results above do not represent the final outcome. Results for CORE-10 at this stage are not an accurate reflection of the service received on board the ManVan because the ‘full set’ is not complete without ‘assessment’, ‘first’, ‘midpoint’, and ‘discharge’ forms.

## Visitors’ views

I saw the van on the road and thought I’d pop in. The team cleared up my questions and provided me with info that is not easily available from elsewhere.

The best thing about the ManVan was learning about the best ways to check for testicular cancer.

I’ve had prostate cancer for 3 years and I’ve learned more about it from you in this session than I was ever told at the hospital.

The visit was excellent. Everyone was professional and approachable.

My dad has recently been diagnosed with prostate cancer, it’s really early stage and non-aggressive so he’s lucky I guess. Just wanted to say thank you, he visited the ManVan, who supported him in getting a test from his GP (who was previously reluctant to do so) and then by complete chance were outside the hospital on the day he had his first appointment. Our experience was the doctor didn’t really want to discuss the emotional aspect of dad’s diagnosis, the consultation was brief and we came out with no more knowledge or plan going forward than when we went in. Thankfully …. The ManVan team spent time with dad and offered him ongoing support. I can’t speak highly enough ….. amazing, very knowledgeable, practical and reassuring. It’s good to know that there are services available to patients that are as supportive as yours.

## Conclusion

Although there are significant limitations at this stage of the project, including small sample sizes and incomplete data sets, early indications are positive that the ManVan is providing a valuable service to men affected by prostate, testicular, and penile cancer across Wales. We are committed to providing the ManVan service in this form until at least 31 March 2017. However, the cancer landscape is changing. New models of care and after care are required. Cancer is increasingly being recognised as a long-term condition and services need to evolve and adapt to this in order to enable people affected by cancer to live a meaningful, quality life. With newly emerging cancer care needs, socio-demographic change and financial pressures, providing long-term cancer support services in community settings, particularly ones that are mobile like the ManVan, is one way of ensuring we are getting maximum value and impact from our cancer support services.

## Figures and Tables

**Table 1.** CORE-10 categories.

	Category	Score
1	Healthy	0–10
2	Moderate	11–20
3	Moderate severe	21–25
4	Severe	26–40

**Table 2.** Appointments offered and attended and client profile by month.

	Apr	May	Jun	Jul	Aug	Sep	Oct	Nov	Dec	Jan	Feb	Mar	Total
**Appointments offered**	**1**	**7**	**22**	**33**	**45**	**53**	**62**	**75**	**57**	**86**	**124**	**105**	**670**
Appointments attended	*1*	*7*	*20*	*29*	*34*	*42*	*49*	*68*	49	77	119	96	591
**Total clients**	**1**	**7**	**18**	**31**	**23**	**21**	**35**	**30**	**14**	**22**	**31**	**33**	**266**
• New clients	*1*	*7*	*17*	*28*	*18*	*13*	*21*	*12*	7	12	17	8	161
• Existing clients	*0*	*0*	*1*	*3*	*5*	*8*	*14*	*18*	7	10	14	25	105

**Table 3.** Mean positivity and arousal scores before and after visiting the ManVan.

	Before	After
Positivity	4.7	5.0
Arousal	3.6	3.8

**Table 4.** Mean combined score for positivity and arousal before and after visiting the ManVan.

	Before	After	Change
Score	4.2	4.4	0.2

**Table 5.** Combined SES and SEQ domain scores.

Domain	Score after being on the ManVan
Positivity	5.0
Arousal	3.8
Depth	5.4
Smoothness	5.6

**Table 6.** Mean changes in CORE-10 scores between stages.

Assessment Core-10	First session Core-10	Midpoint Core-10	Discharge CORE-10
19.12	19.84	16.0	9.5
